# Association of Urinary Phthalates with Self-Reported Eye Affliction/Retinopathy in Individuals with Diabetes: National Health and Nutrition Examination Survey, 2001–2010

**DOI:** 10.1155/2016/7269896

**Published:** 2015-12-20

**Authors:** Manju Mamtani, Joanne E. Curran, John Blangero, Hemant Kulkarni

**Affiliations:** South Texas Diabetes and Obesity Institute, University of Texas Rio Grande Valley School of Medicine, Brownsville, TX 78520, USA

## Abstract

*Background*. An epidemiological association between exposure to phthalates and type 2 diabetes (T2D) is known. However, the potential role of environmental phthalates in the complications of T2D is unknown. *Methods*. Using data from the National Health and Nutrition Examination Survey (NHANES) 2001–2010, we studied the association of 12 urinary phthalate metabolites with self-reported eye affliction/retinopathy in 1,004 participants with diabetes. Data from retinal imaging was used to validate this outcome. Independence of the phthalates*→*T2D association was studied by adjusting for age, sex, race, marital status, educational attainment, poverty income ratio, physical activity, glycated hemoglobin levels, total serum cholesterol, serum high-density lipoprotein cholesterol, serum triglycerides, blood pressure, duration of diabetes, total calorie intake, and obesity. *Results*. Self-reported eye affliction/retinopathy had 82% accuracy with Cohen's kappa of 0.31 (*p* < 0.001). Urinary mono-n-octyl phthalate (MOP) was independently associated with the likelihood of self-reported eye affliction/retinopathy in subjects with T2D after accounting for all the confounders. This significance of this association was robust to the potential misclassification in cases and controls of retinopathy. Further, a significant dose-response relationship between MOP and self-reported eye affliction/retinopathy was demonstrable. *Conclusions*. We show a novel epidemiological link between the environment and diabetic complications in NHANES 2001–2010 participants.

## 1. Introduction

Evidence for the putative association between exposure to phthalates and risk of type 2 diabetes (T2D) is now increasing. For example, association of urinary phthalate metabolites with T2D has been shown in Swedish [1], Mexican [2], and United States populations [3–6]. It has been reported that rats exposed to the phthalate di-(2-ethylhexyl) phthalate (DEHP) develop reversible hyperglycemia, hypoinsulinemia, and symptoms of diabetes [7]. Similarly, developmental exposure to phthalates is known to be associated with *β*-cell dysfunction and glucose abnormality in rats [8]. There also exists additional evidence implicating phthalate exposure in the pathogenesis of insulin resistance [4, 6, 9–12].

Despite this body of evidence, it is currently unknown whether severity of T2D and likelihood of its complications may also be altered depending on exposure to phthalates. Conceivably, sustained and long-term exposure to phthalates can hasten the diabetic process and thereby precipitate the occurrence of diabetic complications. Thus, it can be conjectured that even after the onset of T2D is triggered, the severity of diabetes, as manifested by its complications, may also be modulated by environmental challenges. Alternatively or concomitantly, it is also plausible that acceleration of progress to retinopathy subsequent to phthalate exposure may be a result of a direct effect on the retina. It is instructive in this regard that DEHP is known to partake in retinal vessel remodeling [13]. Also, dimethyl phthalate (DMP), diethyl phthalate (DEP), and DEHP have been shown to influence the activity of retinal aldolase [14]. However, the relative contribution of phthalate exposure to these two possible mechanisms is currently unknown.

In this study, we hypothesized that phthalate exposure is associated with eye-related complications in subjects with T2D. By choosing participants who already have T2D, we attempted to focus on the direct association of phthalates with ocular complications after T2D sets in. To test this hypothesis, we examined the association of twelve phthalate metabolites in urine with the risk of self-reported eye affliction/retinopathy in the publicly available and nationally representative sample of individuals with diabetes recruited in the National Health and Nutrition Examination Survey (NHANES) 2001–2010.

## 2. Materials and Methods

### 2.1. The NHANES 2001–2010 Dataset

**Figure 1:**
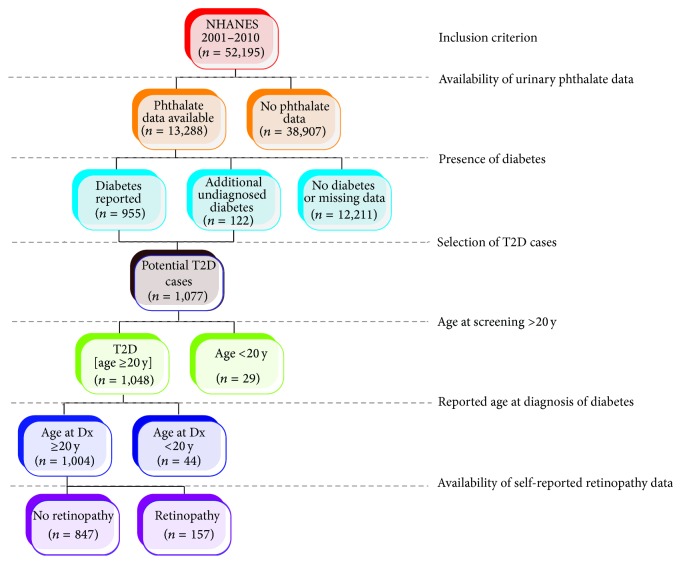
Selection of study subjects. This chart shows how the final number of 1,004 participants included in this study was selected. On the right side are shown the inclusion criteria that correspond to the selection of subjects.

NHANES is an annual survey conducted by the National Center of Health Statistics of the Centers for Disease Control and Prevention (CDC). Detailed description of the NHANES 2001–2010 survey and sampling strategies can be found online at http://www.cdc.gov/nchs/nhanes.htm/. The selection of participants for the present study is schematically shown in Figure 1. In this dataset of 52,195 responders, urinary phthalates were measured in 13,288 (25.5%) individuals. We defined “diabetes” (*n* = 955) as a response of “yes” to one or more of the following questions: “Other than during pregnancy, have you ever been told by a doctor or health professional that you have diabetes or sugar diabetes?” or “Are you now taking insulin?” or “Are you now taking diabetic pills to lower blood sugar?” We excluded individuals who refused to answer the question, did not know the answer, or had a missing value. To maximize the likelihood of capturing participants with diabetes, we also included undiagnosed diabetes cases which were defined as glycated hemoglobin value ≥6.5% [15]. Using this criterion, we could include an additional 122 cases of diabetes. We then proceeded to increase the likelihood that the reported or undiagnosed cases of diabetes are indeed T2D from the 1,077 participants with “diabetes.” We defined T2D as presence of “diabetes” in participants whose age at screening as well as age at diagnosis of diabetes was at least 20 years. Using these criteria, we included a total of 1,004 cases of T2D in this study. These cases were recruited in the NHANES over the 10-year period spanning 2001–2010 and had data on self-reported eye affliction/retinopathy.

### 2.2. Outcome

Self-reported eye affliction/retinopathy was defined as an affirmative answer to the following question: “Has a doctor ever told you that diabetes has affected your eyes or that you had retinopathy?” An estimated 13.87% of the 1,004 study participants reported this outcome. An obvious concern about this outcome was its validity. We examined the validity of this outcome by comparing it with the results of ophthalmologist's reports of detailed eye and retinal examination. The ophthalmological assessment was available only for the NHANES 2005-2006 and NHANES 2007-2008 cycles. These data are based on images of two fundus examinations captured using a nonmydriatic digital camera and the grading was based on the Early Treatment Diabetic Retinopathy Study severity scale [16–18]. The images were graded as no retinopathy, mild nonproliferative retinopathy (NPR), moderate/severe NPR, and proliferative retinopathy (PR). PR was defined as presence of neovascularization on the retinal surface or abnormal growth of new retinal blood vessels into the vitreous.

### 2.3. Estimation of Urinary Phthalate Metabolites

Urinary phthalate metabolites have been measured in a random subsample of NHANES participants (http://www.cdc.gov/exposurereport/ and [19, 20]). Briefly, frozen urine samples (−20°C) were assayed using a combination of solid-phase extraction, high performance liquid chromatography, and tandem mass spectrometry. Concentrations below the corresponding limit of detection (LOD) were replaced by $LOD/\sqrt{2}$ [5]. Although the NHANES 2001–2010 dataset contained information on 15 phthalate metabolites (Table 1), complete measurements on all the included individuals were available for the following 12 phthalate metabolites: mono-n-butyl phthalate (MBP), mono-cyclohexyl phthalate (MCP), mono-ethyl phthalate (MEP), mono-(2-ethyl)-hexyl phthalate (MEHP), mono-isononyl phthalate (MNP), mono-n-octyl phthalate (MOP), mono-benzyl phthalate (MBzP), mono-n-methyl phthalate (MNM), mono-(3-carboxypropyl) phthalate (MCPP), mono-(2-ethyl-5-hydroxyhexyl) phthalate (MEHHP), mono-(2-ethyl-5-oxohexyl) phthalate (MEOHP), and mono-isobutyl phthalate (MiBP). Distribution of the urinary phthalate concentrations in the study participants is shown in Table 1.

### 2.4. Potential Confounders

In addition to urinary concentrations of 12 phthalate metabolites, we considered the following potential confounders: age, sex, race, marital status, educational attainment, poverty income ratio, physical activity (measured as metabolic equivalent hours and categorized as recommended by [21]), glycated hemoglobin levels, total serum cholesterol, serum high-density lipoprotein cholesterol, serum triglycerides, blood pressure, duration of diabetes, total calorie intake (estimated from dietary questionnaires), and obesity (defined on the basis of body mass index, BMI). Distribution of these variables in the study participants and the coding schemes used in analyses are shown in Table 1. To ensure that all the selected study participants are represented in all the multivariable models, we coded the missing values of confounders as zero.

### 2.5. Statistical Analysis

All the analyses were conducted using the Stata 12.0 (StataCorp, College Station, TX) software package. To ensure a normal distribution of urinary phthalate metabolites and the use of a common metric for all the urinary phthalates, we performed a two-step transformation of the urinary concentrations of phthalate metabolites. In the first step, we corrected for urinary dilution by calculating log ratio of the phthalate metabolite concentration and urinary creatinine concentration. In the second step, we inverse-normalized this log ratio by (i) ranking the values, (ii) creating a cumulative density function based on the ranks, and (iii) using the invnorm() function in Stata to create a variable distributed as *N*(0,1). The histograms of raw values, values corrected for dilution, and the inverse-normalized values for each urinary phthalate metabolite are shown in Figures  S1–S15 in Supplementary Material available online at http://dx.doi.org/10.1155/2016/7269896.

To examine the validity of self-reported retinopathy, we estimated Cohen's kappa. To assess the robustness of the observed associations with self-reported eye affliction/retinopathy, we ran 5000 replicates using Monte Carlo simulations. In these simulations, we applied the misclassification rates observed in the validity subsample to the whole sample and estimated the association using the full logistic regression model that included all the covariates mentioned earlier. The Stata program listing of the simulation program is given in Figure  S16.

We used the svy set of commands to adjust for the sampling weights and design variables as recommended by the Centers for Disease Control (http://www.cdc.gov/nchs/tutorials/). Descriptive statistics such as means and proportions were also adjusted for the survey design variables. The association analyses employed univariate and multivariable logistic regression and were conducted using the logistic subcommand of the svy command. For the univariate analyses, we corrected the significance values for multiple comparisons using the Bonferroni method. Hypothesis of for dose-response relationship was tested using the Armitage test for linear trend. Statistical significance was evaluated at a global type I error rate of 0.05.

## 3. Results

### 3.1. Association of Urinary Phthalate Metabolites with Self-Reported Eye Affliction/Retinopathy

When each phthalate metabolite was separately regressed on the study outcome in a univariate fashion (Table 2), only MOP was significantly associated with eye affliction/retinopathy such that one standard deviation increase in urinary MOP was associated with a 1.39 times higher likelihood (Bonferroni-corrected *P* = 0.048) of eye affliction/retinopathy in participants with diabetes.

To test the robustness of this observation, we first conducted a series of multivariable analyses since there was a complex pattern of correlations among the 12 phthalate metabolites (Table S1). For the multivariable analyses, we conducted a forward stepwise logistic regression retaining all the included covariates in each step. These results are shown in Table 3. Firstly we included all the 12 urinary phthalates as covariates and found that MOP was significantly associated with the study outcome. Stepwise addition of all the other demographic and clinical variables did not influence the independent association of MOP. The results from the final model including all 26 covariates in addition to MOP are shown in Table S2. The final model identified three significant predictors of self-reported eye affliction/retinopathy: MOP (OR 2.02, 95% CI 1.22–3.35), low HDL cholesterol (OR 0.54, 95% CI 0.33–0.89), and duration of diabetes (OR 1.95, 95% CI 1.62–2.33).

### 3.2. Validation of Self-Reported Retinopathy/Eye Affliction

Data on self-reported eye affliction/retinopathy and ophthalmological examination was available on 285 participants with T2D (Table 4). In these participants, the prevalence of mild nonproliferative retinopathy, moderate/severe nonproliferative retinopathy, and proliferative retinopathy was 23.8, 7.7, and 1.6%, respectively. The proportion of self-reported eye affliction/retinopathy was 11%, 15%, 46%, and 48% in participants who had no retinopathy, mild NPR, moderate/severe NPR, and PR, respectively.

There was an 82.01% agreement between these two outcomes which translated into a Cohen's kappa of 0.31 (*P* = 1.2 × 10^−7^). As evidenced from the results shown in Table 4, 19 participants (design-corrected proportion 4.92%) who had moderate/severe NPR or PR were missed by self-reported retinopathy/eye affliction indicating possible underreporting. On the other hand, there were 33 (design-corrected proportion 11.2%) participants who reported eye affliction/retinopathy but did not have corroborative imaging data.

**Figure 2:**
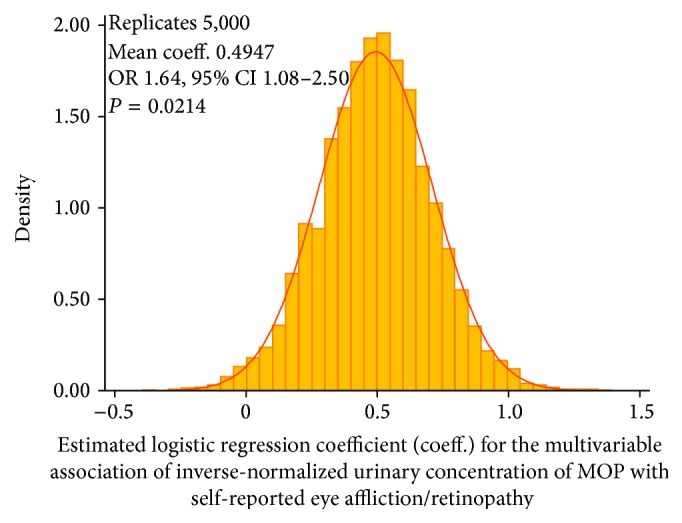
Monte Carlo simulations of the association of MOP with diabetic retinopathy after applying the misclassification rates observed in the validation sample. This plot shows the histogram of 5,000 logistic regression coefficients with an overlaid normal curve. All models use inverse-normalized concentrations of phthalate metabolites. OR, odds ratio; CI, confidence interval; *P*, significance value.

Since ophthalmologically determined retinopathy was available in only 285 participants, the full set of association analyses could not be run on this smaller subsample. Instead, we generated 5000 replicates using a Monte Carlo procedure in which the cases and controls of self-reported eye affliction/retinopathy were randomly reshuffled based on the misclassification rates (11.2% and 4.92%) observed in the validation subsample. For each of these replicates, we ran the full logistic regression model shown in Table 3 (Model 16) and determined distribution of the regression coefficient for the association of inverse-normalized MOP with self-reported eye affliction/retinopathy. We observed (Figure 2) that the estimated regression coefficient followed a normal distribution with a mean of 0.4947 which translates to an OR of 1.64 (95% CI 1.08–2.50). These results imply that even if one accounts for possible errors of misclassifying of cases and controls of retinopathy due to self-reporting, the association of MOP would still be significant.

### 3.3. Dose-Response Relationship of Urinary MOP with Self-Reported Eye Affliction/Retinopathy

**Figure 3:**
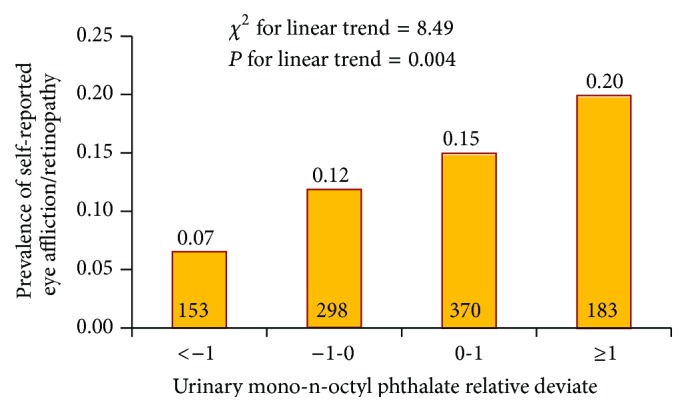
Dose-response relationship of urinary mono-n-octyl phthalate with self-reported eye affliction/retinopathy in NHANES 2001–2010 participants. Bars represent the survey-design-corrected prevalence within categories of subjects defined by the relative deviate (mean/standard deviation) of inverse-normalized, dilution corrected urinary MOP concentration. Linear trend was assessed using the Armitage test for linear trend.

We next examined if there was evidence for a dose-response relationship of the inverse-normalized urinary MOP concentration and prevalence of eye affliction/retinopathy. We found that indeed there was a clearly significant linear increase in the prevalence based on the dose of MOP (Figure 3). The prevalence of self-reported eye affliction/retinopathy steadily increased from 0.07 (in participants with >1 SD below mean MOP) to 0.21 (in participants with >1 SD above mean MOP). Armitage test for linear trend showed a significantly linear association (*P* = 0.004) between urinary MOP and self-reported retinopathy/eye affliction.

### 3.4. Specificity of Association of MOP with Self-Reported Eye Affliction/Retinopathy

Since MOP is a rarer metabolite of DnOP, we investigated whether the observed concentrations were confounded by the ability of the assay to detect this metabolite in urine. We found that in 73% of the participants included in this study the urinary concentration of MOP was above the lower limit of detection (0.84 ng/mL). Secondly, we created a variable to capture the molar sum of DnOP derivatives (MOP and MCPP) and tested the association of the molar sum of DnOP with self-reported eye affliction/retinopathy. We found that this association was not statistically significant (OR 0.92, 95% CI 0.76–1.12, *P* = 0.393).

## 4. Discussion

### 4.1. What Do These Results Mean?

Our results indicate a specific, robust, independent, and statistically significant association of urinary MOP concentration with the likelihood of self-reported eye affliction/retinopathy in a nationally representative sample of participants with diabetes from the United States. This is a novel observation. Interestingly, of the three metabolites of DEHP, MEHP, MEHHP, and MEOHP [5], none showed a statistically trending association (in the multivariable adjusted model). Since MOP is a metabolite of DnOP [22], our study has identified exposure to DnOP as a potentially important contributor to ocular complications of diabetes. It is intriguing, however, that the other metabolite of DnOP (MCPP) was not significantly associated with self-reported eye affliction/retinopathy. In the metabolism of DnOP, MOP is the result of the first step of hydrolysis while MCPP is the result of the next step of oxidation. It is conceivable, for example, that the enzymatic progression from the stage of hydrolysis to oxidation may be dysfunctional or subfunctional in DR resulting in an increase in MOP without significantly affecting the concentration of the downstream metabolite (MCPP). Direct* in vitro* and* ex vivo* studies need to be undertaken to demonstrate these postulated perturbations in the metabolic pathway of phthalates but our results point towards this interesting and putative hypothesis. Our results also indicate that phthalate exposure may be associated with not only an increased likelihood of T2D but also its complications. Notably, our sample included non-DR diabetic patients as controls and, therefore, the observed associations are indicative of an epidemiological link between DR and MOP rather than between underlying diabetes and MOP. While making these and other conclusions from this study, it must be considered that these results only provide associative evidence and therefore causal conclusions need to be refrained from.

It is also noteworthy that we observed an association of low HDL cholesterol with self-reported retinopathy. It is generally believed that low HDL is not associated with diabetic retinopathy [23–25]. However, evidence to the contrary also exists from several populations around the world. For example, in a study by Sasongko et al. [26], it was found that of all the routinely used lipid measures, low HDL provided the most significant and independent contribution to prediction of DR. Other studies from United States [27], China [28], and India [29] also provide supportive evidence to the association of low HDL and DR. It is instructive, in this regard, that the global multicentric case-control study of DR [30] and the Italian RIACE study [31] posit that the observed association between low HDL and DR can be partly explained on the basis of coexisting chronic kidney complications of diabetes. We did not have data on coexisting diabetic nephropathy and therefore cannot comment on the possibility of a nephropathy driven association between low HDL and DR. Together, published literature does not offer a definitive answer on the association of low HDL with DR. Future studies need to carefully address this issue using a combination of meta-analytical and mechanistic approaches.

### 4.2. Biological Plausibility

If indeed MOP is a player in the complex web of diabetic retinopathy then a biological rationale for such a finding deserves consideration. Diabetic retinopathy typically results in neovascularization of retinal vessels with a consequent bleeding into the vitreous and/or macular edema. This process has a strong genetic basis. In an elegant recent review, Murea et al. [32] enlisted eight genes that have been previously implicated in the pathogenesis of diabetic retinopathy. Phthalates have been shown to alter expression of or signaling through several of these genes [33–40] and one of these genes partakes in the pathway orchestrated by the peroxisome proliferator-activated receptor gamma gene (*PPARγ*), a known target of all phthalates including MOP. Thus, there may be a genetic basis to the possible precipitation of diabetic retinopathy by exposure to MOP.

Also, using an* in silico* approach, Jananie et al. [41] recently demonstrated that phthalates can bind angiotensin II type 1 (AT1) receptor which plays a central role in diabetic retinopathy. Action of AT1 in the retinal microvessels and pericytes can contribute to diabetic retinopathy [42–44]. Whether this mechanism is operational in exposure to MOP is unknown and needs to be investigated in future studies. However, the plethora of animal and human studies that point towards a consistent association of some phthalates with retinopathy provide an indirect (and, admittedly, insufficient) evidence for a biological basis to the association of MOP with diabetic retinopathy observed in this study. The possible mechanisms outlined here need to be considered in future studies.

### 4.3. Limitations

Our study had three limitations. First, the outcome was self-reported. Although recall bias can be expected to be low for this outcome, such a bias cannot be eliminated. Validation of this outcome based on retinal imaging studies also indicated that some misclassification was evident. However, our analyses using Monte Carlo simulation indicate that the potential misclassification due to an alloyed outcome used in this study is unlikely to negate the association of MOP with diabetic retinopathy. Second, urinary concentrations of phthalate metabolites only indirectly imply environmental exposure [6]. The pattern, dose, and modes of actual exposure to phthalates cannot be discerned from this study. Thus, whether the associations reflect a cumulative risk due to sustained exposure cannot be reliably answered from these data. Also, currently there is limited data available on the sources of phthalate exposure. Third, since the temporal sequence of phthalate exposure and complications of diabetes is unknown, the evidence presented here can at best be considered associative and not causal.

## 5. Conclusions

Urinary phthalate concentrations are considered to be a reasonable biomarker of chronic exposure to phthalates [45]. Since the biological effects of phthalate exposure are likely reversible [7], our results beckon a critical and intensive look into the potential role of MOP in diabetic retinopathy. The ubiquity of DnOP in products like flooring tiles and cosmetics [46] entails that future studies need to carefully dissect out the putative epidemiological link observed in this study.

## Supplementary Material

The Supplementary Figures S1–S15 show the effect of statistical transformation of the urinary concentrations of 15 phthalate metabolites. Figures show effect of correction for dilution and of inverse-normalization on the distribution of the indicated phthalate metabolites concentrations in urine. Figure S16 shows a Stata program listing that was used to conduct Monte Carlo simulations of misclassification rates based on a validation sample. Supplementary Table S1 shows the lower triangular part of the correlation matrix that summarizes pairwise correlations among all the phthalate metabolites. Supplementary Table S2 shows the full results of the logistic regression model described in Table 3 of the main text.

## Figures and Tables

**Table 1:** Outcome and potential confounders considered in the analyses.

Variable	Value	Code
Outcome		
Self-reported retinopathy [*n* (%)]		
No	157 (13.87)	0
Yes	847 (86.13)	1
Demographic variables		
Age [mean (SE)]^†^ y	60.12 (0.56)	Raw
Sex [*n* (%)^††^]		
Females	483 (50.6)	0
Males	521 (49.4)	1
Ethnicity [*n* (%)]		
Mexican Americans	215 (7.86)	1
Hispanics	72 (4.92)	1
Non-Hispanic Whites	415 (65.65)	0
Non-Hispanic Blacks	267 (16.07)	0
Other non-Hispanics	35 (5.50)	0
Marital status [*n* (%)]		
Married	555 (58.64)	1
Others^*∗*^	449 (41.36)	0
Educational attainment [*n* (%)]		
Less than 9th Grade	216 (12.73)	1
9–11th Grade	191 (16.21)	2
High school grad/GED or equivalent	219 (22.51)	3
Some college or AA degree	253 (30.08)	4
College graduate or above	124 (18.44)	5
Refused or unknown	1 (0.03)	0
Poverty income ratio [mean (SE)]	2.87 (0.07)	0 if unknown, or else raw
Clinical variables		
Total calorie intake per day [*n* (%)]		
Q1 (<1,200.5 kcal)	251 (20.41)	0
Q2 (1,200.5–<1,648.5 kcal)	251 (23.60)	1
Q3 (1,648.5–<2,191 kcal)	251 (26.02)	2
Q4 (≥2,191 kcal)	251 (29.97)	3
Obesity		
BMI ≥30 Kg/m^2^	559 (60.41)	2
BMI <30 Kg/m^2^	416 (37.03)	1
BMI not measured	29 (2.55)	0
Physical activity categories		
Active (>6 METS)	168 (20.50)	2
Insufficiently active (3–6 METS)	350 (37.14)	1
Inactive (<3 METS) or unknown	486 (42.36)	0
Glycated hemoglobin [*n* (%)]		
≥6.5%	433 (42.17)	2
<6.5%	178 (18.33)	1
Unknown	393 (39.51)	0
High serum total cholesterol [*n* (%)]		
≥240 mg/dL	129 (14.02)	2
<240 mg/dL	814 (81.19)	1
Unknown	129 (4.79)	0
Low HDL cholesterol [*n* (%)]		
Males <40 mg/dL, females <50 mg/dL	385 (42.82)	2
Males ≥40 mg/dL, females ≥50 mg/dL	559 (52.58)	1
Unknown	60 (4.60)	0
High serum triglycerides [*n* (%)]		
≥150 mg/dL	220 (23.73)	2
<150 mg/dL	258 (24.65)	1
Unknown	526 (51.61)	0
Hypertension [*n* (%)]		
Yes	305 (28.39)	2
No	654 (67.04)	1
Unknown	45 (4.57)	0
Duration of diabetes [*n* (%)]		
≥14 y	225 (19.06)	4
8–13 y	190 (18.96)	3
3–7 y	242 (25.54)	2
<3 y	172 (19.04)	1
Unknown	175 (17.38)	0
Urinary phthalates, ng/mL [mean (SE)]		
Mono-n-butyl (MBP)	47.30 (11.87)	Corrected for urinary creatinine and inverse-normalized
Mono-cyclohexyl (MCP)	0.41 (0.02)	
Mono-ethyl (MEP)	464.77 (47.07)	
Mono-(2-ethyl)-hexyl (MEHP)	6.44 (0.93)	
Mono-isononyl (MNP)	1.54 (0.17)	
Mono-n-octyl (MOP)	1.13 (0.02)	
Mono-benzyl (MBzP)	12.59 (0.75)	
Mono-n-methyl (MNM)	3.85 (0.44)	
Mono-(3-carboxypropyl) (MCPP)	5.38 (0.49)	
Mono-(2-ethyl-5-hydroxyhexyl) (MEHHP)	42.34 (4.12)	
Mono-(2-ethyl-5-oxohexyl) (MEOHP)	25.66 (2.42)	
Mono-isobutyl (MiBP)	9.00 (0.80)	
Mono-2-ethyl-5-carboxypentyl (MECPP)^#^	58.72 (5.56)	Not used
Mono-(carboxynonyl) (MCNP)^##^	4.37 (0.35)	Not used
Mono-(carboxyoctyl) (MCOP)^##^	20.40 (2.41)	Not used

^†^All means and standard errors are adjusted for survey design variables.

^††^All proportions are adjusted for survey design variables.

^*∗*^Widowed, divorced, separated, never married, living with partner, and refused.

^#^Data available on 828 participants.

^##^Data available on 649 participants.

**Table 2:** Univariate association of urinary phthalate metabolites with self-reported eye affliction/retinopathy in participants with diabetes, NHANES 2001–2010^†,*∗*^.

Phthalate	OR	95% CI	*P* ^*∗∗*^	*P*_c_^*∗∗*^
Mono-n-butyl (MBP)	1.12	0.90–1.39	0.285	1.000
Mono-cyclohexyl (MCP)	1.28	0.99–1.63	0.053	0.636
Mono-ethyl (MEP)	1.08	0.83–1.40	0.583	1.000
Mono-(2-ethyl)-hexyl (MEHP)	1.02	0.83–1.25	0.860	1.000
Mono-isononyl (MNP)	1.19	0.95–1.49	0.120	1.000
Mono-n-octyl (MOP)	1.39	1.11–1.74	0.004	0.048
Mono-benzyl (MBzP)	1.20	0.91–1.57	0.196	1.000
Mono-n-methyl (MNM)	1.10	0.85–1.42	0.455	1.000
Mono-(3-carboxypropyl) (MCPP)	1.01	0.83–1.24	0.893	1.000
Mono-(2-ethyl-5-hydroxyhexyl) (MEHHP)	1.17	0.93–1.46	0.176	1.000
Mono-(2-ethyl-5-oxohexyl) (MEOHP)	1.22	0.97–1.54	0.084	1.000
Mono-isobutyl (MiBP)	1.14	0.90–1.45	0.278	1.000

^†^All models account for the survey design variables using svy command in Stata and use inverse-normalized concentrations of phthalate metabolites.

^*∗*^Results are from separate logistic regression models for each phthalate metabolite.

^*∗∗*^ *P*: nominal significance value; *P*_c_: Bonferroni-corrected significance value.

**Table 3:** Multivariable association of MOP with self-reported eye affliction/retinopathy through nested logistic regression models, NHANES 2001–2010^*∗*^.

Model	Covariates	OR	95% CI	*P*
1	MBP, MEP, MCP, MEHP, MNP, MBzP, MNM, MCPP, MEHHP, MEOHP, and MiBP	2.11	1.18–3.77	0.013
2	Model 1 and age	2.11	1.17–3.81	0.014
3	Model 2 and sex	2.13	1.19–3.81	0.012
4	Model 3 and Hispanic/Mexican race	2.13	1.18–3.82	0.012
5	Model 4 and marital status	2.13	1.19–3.79	0.011
6	Model 5 and educational attainment	2.09	1.18–3.68	0.012
7	Model 6 and poverty income ratio	2.09	1.18–3.68	0.012
8	Model 7 and physical activity	2.11	1.20–3.71	0.010
9	Model 8 and HbA1c strata	2.02	1.16–3.49	0.013
10	Model 9 and total serum cholesterol strata	2.00	1.19–3.37	0.010
11	Model 10 and HDL cholesterol strata	2.01	1.21–3.32	0.007
12	Model 11 and serum triglycerides strata	2.01	1.21–3.33	0.007
13	Model 12 and hypertension	2.01	1.22–3.32	0.007
14	Model 13 and duration of diabetes	2.03	1.22–3.39	0.007
15	Model 14 and quartiles of total calorie intake per day	2.03	1.22–3.38	0.007
16	Model 15 and obesity	2.02	1.22–3.35	0.007

^*∗*^All models account for the survey design variables using svy command in Stata and use inverse-normalized concentrations of phthalate metabolites; covariate definitions are provided in Table 1.

**Table 4:** Comparison of self-reported retinopathy in a subsample (*n* = 285) of NHANES 2001–2010 data.

Retinopathy level by imaging	Self-reported retinopathy		Design-corrected proportion (%)
	No	Yes	
*Compared to 4-level retinopathy*			
No retinopathy	157	22	11.31
Mild NPR	59	11	15.27
Moderate/severe NPR	15	13	46.33
PR	4	4	48.50

*Compared to presence of moderate or severe retinopathy*			
No/mild NPR	216	33	12.34
Moderate/severe NPR or PR	19	17	46.70

NPR: nonproliferative retinopathy; PR: proliferative retinopathy.

## References

[1] Lind P. M., Zethelius B., Lind L. (2012). Circulating levels of phthalate metabolites are associated with prevalent diabetes in the elderly. *Diabetes Care*.

[2] Svensson K., Hernández-Ramírez R. U., Burguete-García A. (2011). Phthalate exposure associated with self-reported diabetes among Mexican women. *Environmental Research*.

[3] Huang T., Saxena A. R., Isganaitis E., James-Todd T. (2014). Gender and racial/ethnic differences in the associations of urinary phthalate metabolites with markers of diabetes risk: national health and nutrition examination survey 2001–2008. *Environmental Health*.

[4] Trasande L., Spanier A. J., Sathyanarayana S., Attina T. M., Blustein J. (2013). Urinary phthalates and increased insulin resistance in adolescents. *Pediatrics*.

[5] James-Todd T., Stahlhut R., Meeker J. D. (2012). Urinary phthalate metabolite concentrations and diabetes among women in the national health and nutrition examination survey (NHANES) 2001–2008. *Environmental Health Perspectives*.

[6] Stahlhut R. W., van Wijngaarden E., Dye T. D., Cook S., Swan S. H. (2007). Concentrations of urinary phthalate metabolites are associated with increased waist circumference and insulin resistance in adult U.S. Males. *Environmental Health Perspectives*.

[7] Gayathri N. S., Dhanya C. R., Indu A. R., Kurup P. A. (2004). Changes in some hormones by low doses of di (2-ethyl hexyl) phthalate (DEHP), a commonly used plasticizer in PVC blood storage bags & medical tubing. *The Indian Journal of Medical Research*.

[8] Lin Y., Wei J., Li Y. (2011). Developmental exposure to di(2-ethylhexyl) phthalate impairs endocrine pancreas and leads to long-term adverse effects on glucose homeostasis in the rat. *American Journal of Physiology—Endocrinology and Metabolism*.

[9] Hatch E. E., Nelson J. W., Stahlhut R. W., Webster T. F. (2010). Association of endocrine disruptors and obesity: perspectives from epidemiological studies. *International Journal of Andrology*.

[10] Rajesh P., Sathish S., Srinivasan C., Selvaraj J., Balasubramanian K. (2013). Phthalate is associated with insulin resistance in adipose tissue of male rat: role of antioxidant vitamins. *Journal of Cellular Biochemistry*.

[11] Boberg J., Metzdorff S., Wortziger R. (2008). Impact of diisobutyl phthalate and other PPAR agonists on steroidogenesis and plasma insulin and leptin levels in fetal rats. *Toxicology*.

[12] Teitelbaum S. L., Mervish N., L. Moshier E. (2012). Associations between phthalate metabolite urinary concentrations and body size measures in New York City children. *Environmental Research*.

[13] Zei D., Pascarella A., Barrese C., Pantalone S., Stefanini S. (2009). DEHP effects on retinal vessels in newborn rats: a qualitative and quantitative analysis. *Histochemistry and Cell Biology*.

[14] Askari S. N., Zaidi M., Ahmad N. (2006). In-vitro effect of phthalate esters on retinal aldolase. *Turkish Journal of Medical Sciences*.

[15] The International Expert Committee (2009). International Expert Committee report on the role of the A1C assay in the diagnosis of diabetes. *Diabetes Care*.

[16] Zhang X., Cotch M. F., Ryskulova A. (2012). Vision health disparities in the United States by race/ethnicity, education, and economic status: findings from two nationally representative surveys. *American Journal of Ophthalmology*.

[17] Zhang X., Saaddine J. B., Chou C.-F. (2010). Prevalence of diabetic retinopathy in the United States, 2005–2008. *Journal of the American Medical Association*.

[18] ETDRS (1991). Fundus photographic risk factors for progression of diabetic retinopathy. ETDRS report number 12. Early Treatment Diabetic Retinopathy Study Research Group. *Ophthalmology*.

[19] Hauser R., Calafat A. M. (2005). Phthalates and human health. *Occupational and Environmental Medicine*.

[20] Silva M. J., Slakman A. R., Reidy J. A. (2004). Analysis of human urine for fifteen phthalate metabolites using automated solid-phase extraction. *Journal of Chromatography B: Analytical Technologies in the Biomedical and Life Sciences*.

[21] Pate R. R., Pratt M., Blair S. N. (1995). Physical activity and public health. A recommendation from the Centers for Disease Control and Prevention and the American College of Sports Medicine. *Journal of the American Medical Association*.

[22] Carlson K. R. (2010). *Toxicity Review of Di-n-octyl Phthalate*.

[23] Klein B. E. K., Moss S. E., Klein R., Surawicz T. S. (1991). The Wisconsin Epidemiologic Study of Diabetic Retinopathy XIII. Relationship of serum cholesterol to retinopathy and hard exudate. *Ophthalmology*.

[24] Morton J., Zoungas S., Li Q. (2012). Low HDL cholesterol and the risk of diabetic nephropathy and retinopathy: results of the advance study. *Diabetes Care*.

[25] Klein B. E., Myers C. E., Howard K. P., Klein R. (2015). Serum lipids and proliferative diabetic retinopathy and macular edema in persons with long-term type 1 diabetes mellitus: the Wisconsin epidemiologic study of diabetic retinopathy. *JAMA Ophthalmology*.

[26] Sasongko M. B., Wong T. Y., Nguyen T. T. (2011). Serum apolipoprotein AI and B are stronger biomarkers of diabetic retinopathy than traditional lipids. *Diabetes Care*.

[27] Lyons T. J., Jenkins A. J., Zheng D. (2004). Diabetic retinopathy and serum lipoprotein subclasses in the DCCT/EDIC cohort. *Investigative Ophthalmology & Visual Science*.

[28] Xie X. W., Xu L., Wang Y. X., Jonas J. B. (2008). Prevalence and associated factors of diabetic retinopathy. The Beijing Eye Study 2006. *Graefe's Archive for Clinical and Experimental Ophthalmology*.

[29] Agroiya P., Philip R., Saran S., Gutch M., Tyagi R., Gupta K. (2013). Association of serum lipids with diabetic retinopathy in type 2 diabetes. *Indian Journal of Endocrinology and Metabolism*.

[30] Sacks F. M., Hermans M. P., Fioretto P. (2014). Association between plasma triglycerides and high-density lipoprotein cholesterol and microvascular kidney disease and retinopathy in type 2 diabetes mellitus: a global case-control study in 13 countries. *Circulation*.

[31] Penno G., Solini A., Zoppini G. (2012). Rate and determinants of association between advanced retinopathy and chronic kidney disease in patients with type 2 diabetes: the renal insufficiency and cardiovascular events (RIACE) Italian multicenter study. *Diabetes Care*.

[32] Murea M., Ma L., Freedman B. I. (2012). Genetic and environmental factors associated with type 2 diabetes and diabetic vascular complications. *The Review of Diabetic Studies*.

[33] Li J., Ma M., Wang Z. (2008). A two-hybrid yeast assay to quantify the effects of xenobiotics on retinoid X receptor-mediated gene expression. *Toxicology Letters*.

[34] Grün F., Blumberg B. (2007). Perturbed nuclear receptor signaling by environmental obesogens as emerging factors in the obesity crisis. *Reviews in Endocrine & Metabolic Disorders*.

[35] Hsieh T.-H., Tsai C.-F., Hsu C.-Y. (2012). N-butyl benzyl phthalate promotes breast cancer progression by inducing expression of lymphoid enhancer factor 1. *PLoS ONE*.

[36] Just A. C., Whyatt R. M., Miller R. L. (2012). Children's urinary phthalate metabolites and fractional exhaled nitric oxide in an Urban cohort. *American Journal of Respiratory and Critical Care Medicine*.

[37] Vetrano A. M., Laskin D. L., Archer F. (2010). Inflammatory effects of phthalates in neonatal neutrophils. *Pediatric Research*.

[38] Buteau-Lozano H., Velasco G., Cristofari M., Balaguer P., Perrot-Applanat M. (2008). Xenoestrogens modulate vascular endothelial growth factor secretion in breast cancer cells through an estrogen receptor-dependent mechanism. *Journal of Endocrinology*.

[39] Huang P.-C., Tsai E.-M., Li W.-F. (2010). Association between phthalate exposure and glutathione S-transferase M1 polymorphism in adenomyosis, leiomyoma and endometriosis. *Human Reproduction*.

[40] Park H. Y., Kim J. H., Lim Y.-H., Bae S., Hong Y.-C. (2013). Influence of genetic polymorphisms on the association between phthalate exposure and pulmonary function in the elderly. *Environmental Research*.

[41] Jananie R. K., Priya V., Vijayalakshmi K. (2012). Secondary metabolites of *Cynodon dactylon* as an antagonist to angiotensin II type1 receptor: novel *in silico* drug targeting approach for diabetic retinopathy. *Journal of Pharmacology and Pharmacotherapeutics*.

[42] Satofuka S., Kanda A., Ishida S. (2012). Receptor-associated prorenin system in the pathogenesis of retinal diseases. *Frontiers in Bioscience*.

[43] Jeganathan V. S. E. (2011). The therapeutic implications of renin-angiotensin system blockade in diabetic retinopathy. *Current Pharmaceutical Biotechnology*.

[44] Gao B.-B., Phipps J. A., Bursell D., Clermont A. C., Feener E. P. (2009). Angiotensin AT1 receptor antagonism ameliorates murine retinal proteome changes induced by diabetes. *Journal of Proteome Research*.

[45] Hauser R., Meeker J. D., Park S., Silva M. J., Calafat A. M. (2004). Temporal variability of urinary phthalate metabolite levels in men of reproductive age. *Environmental Health Perspectives*.

[46] Calafat A. M., Silva M. J., Reidy J. A. (2006). Mono-(3-carboxypropyl) phthalate, a metabolite of di-n-octyl phthalate. *Journal of Toxicology and Environmental Health Part A*.

