# Supplementary material for: Association of Urinary Phthalates with Self-Reported Eye Affliction/Retinopathy in Individuals with Diabetes: National Health and Nutrition Examination Survey, 2001–2010
Source: J Diabetes Res. 2015 Dec 20;2016:7269896. doi: 10.1155/2016/7269896 (PMC4698956; doi:10.1155/2016/7269896)
Supplement: Supplementary file 1 — The Supplementary Figures S1–S15 show the effect of statistical transformation of the urinary concentrations of 15 phthalate metabolites. Figures show effect of correction for dilution and of inverse-normalization on the distribution of the indicated phthalate metabolites concentrations in urine. Figure S16 shows a Stata program listing that was used to conduct Monte Carlo simulations of misclassification rates based on a validation sample. Supplementary Table S1 shows the lower triangular part of the correlation matrix that summarizes pairwise correlations among all the phthalate metabolites. Supplementary Table S2 shows the full results of the logistic regression model described in Table 3 of the main text. [file 7269896.f1.pdf]

SUPPLEMENTARY FIGURES

FOR

ASSOCIATION OF URINARY PHTHALATES WITH SELF-REPORTED EYE AFFLICTION/RETINOPATHY IN INDIVIDUALS WITH DIABETES: NATIONAL HEALTH AND NUTRITION EXAMINATION SURVEY, 2001-2010

Manju Mamtani, MD, Joanne E. Curran, PhD, John Blangero, PhD, Hemant Kulkarni, MD

CONTENTS

This file shows the effect of transformation on the distribution of urinary phthalate metabolites. For each metabolite, we first show the effect of correction for dilution (panels titled "Corrected for dilution") and then of inverse-normalization (panels titled "Inverse-normalized"). For details of transformation methods, please see Methods section in main text.

Figure	Phthalate metabolite	Page
S1	Mono-n-butyl phthalate (MBP)	2
S2	Mono-cyclohexyl phthalate (MCP)	3
S3	Mono-ethyl phthalate (MEP)	4
S4	Mono-(2-ethyl)-hexyl phthalate (MEHP)	5
S5	Mono-isononyl phthalate (MNP)	6
S6	Mono-n-octyl phthalate (MOP)	7
S7	Mono-benzyl phthalate (MBzP)	8
S8	Mono-n-methyl phthalate (MNM)	9
S9	Mono-(3-carboxypropyl) phthalate (MCPP)	10
S10	Mono-(2-ethyl-5-hydroxyhexyl) phthalate (MEHHP)	11
S11	Mono-(2-ethyl-5-oxohexyl) phthalate (MEOHP)	12
S12	Mono-isobutyl phthalate (MiBP)	13
S13	Mono-2-ethyl-5-carboxypentyl phthalate (MECPP)	14
S14	Mono-(carboxynonyl) phthalate (MCNP)	15
S15	Mono-(carboxyoctyl) phthalate (MCOP)	16
S16	Program listing for Monte Carlo simulations	17

Figure S1: Mono-n-butyl phthalate (MBP)

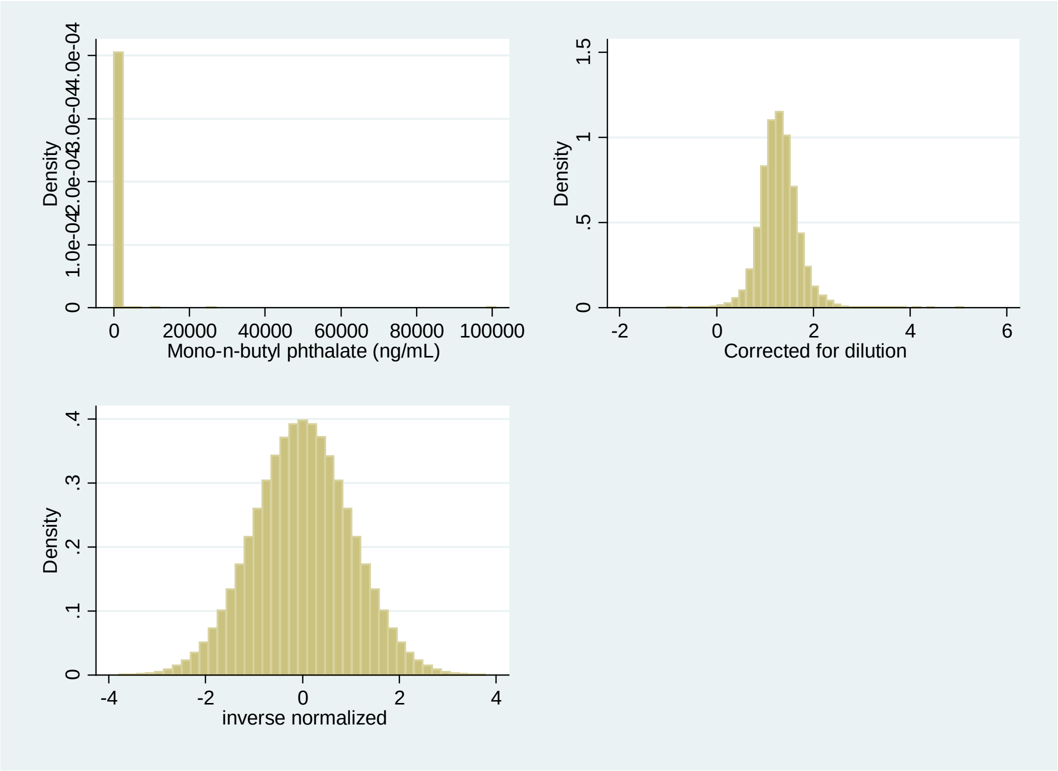

Figure S2: Mono-cyclohexyl phthalate (MCP)

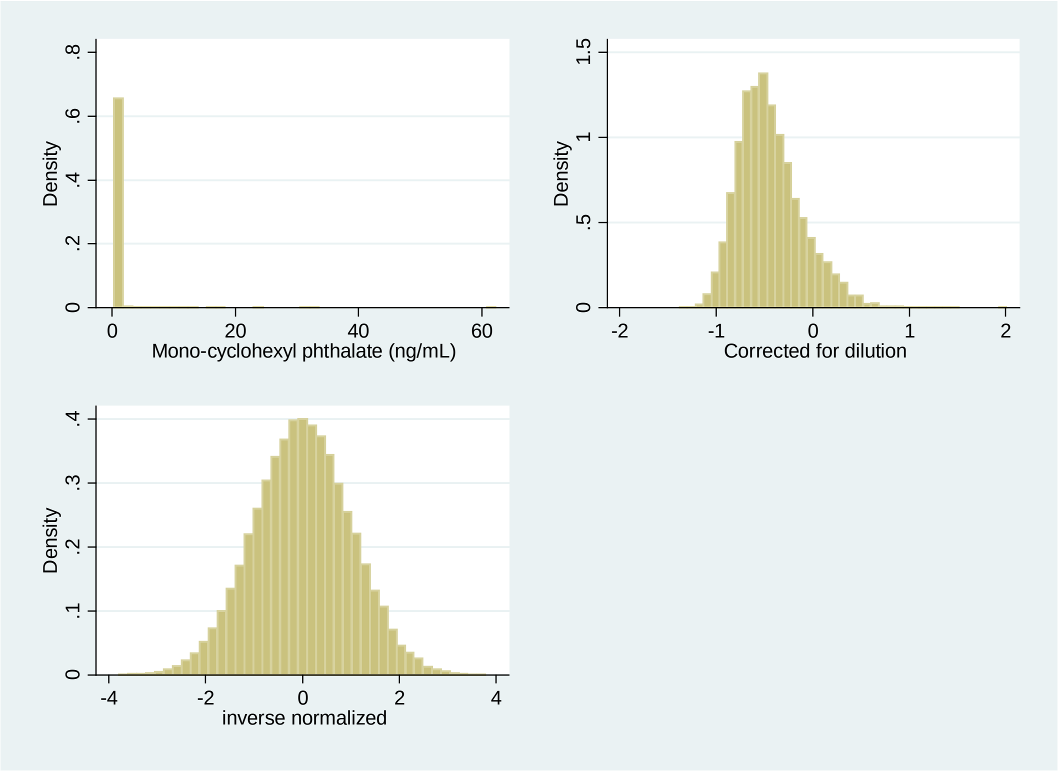

Figure S3: Mono-ethyl phthalate (MEP)

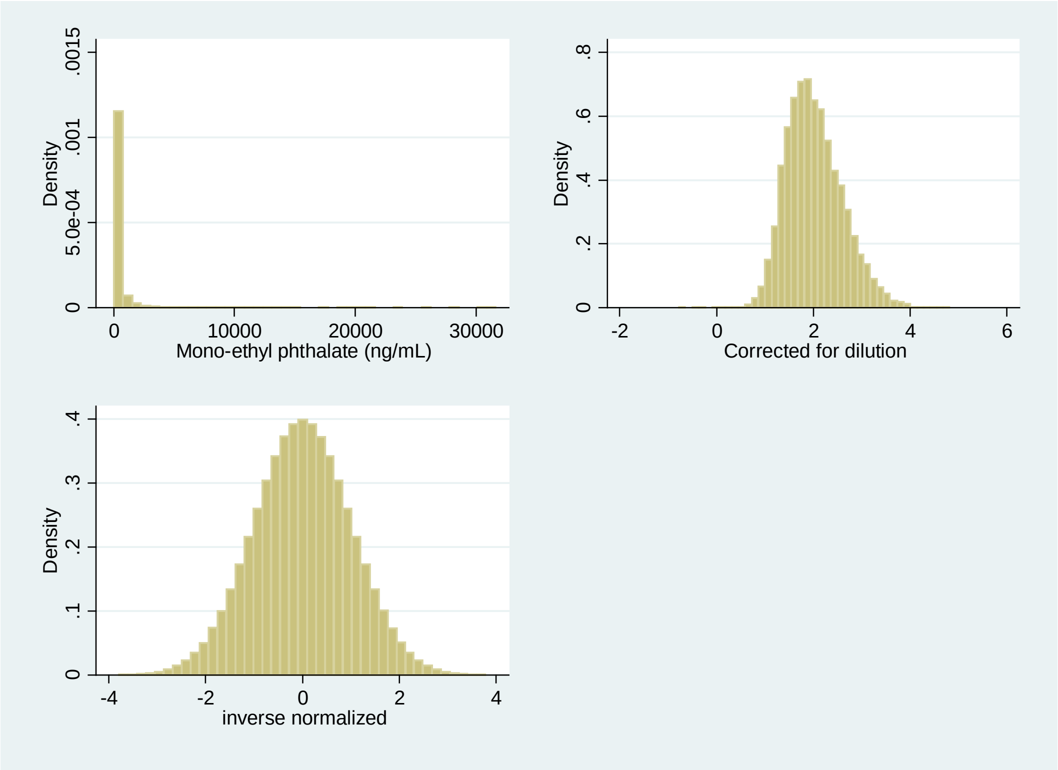

Figure S4: Mono-(2-ethyl)-hexyl phthalate (MEHP)

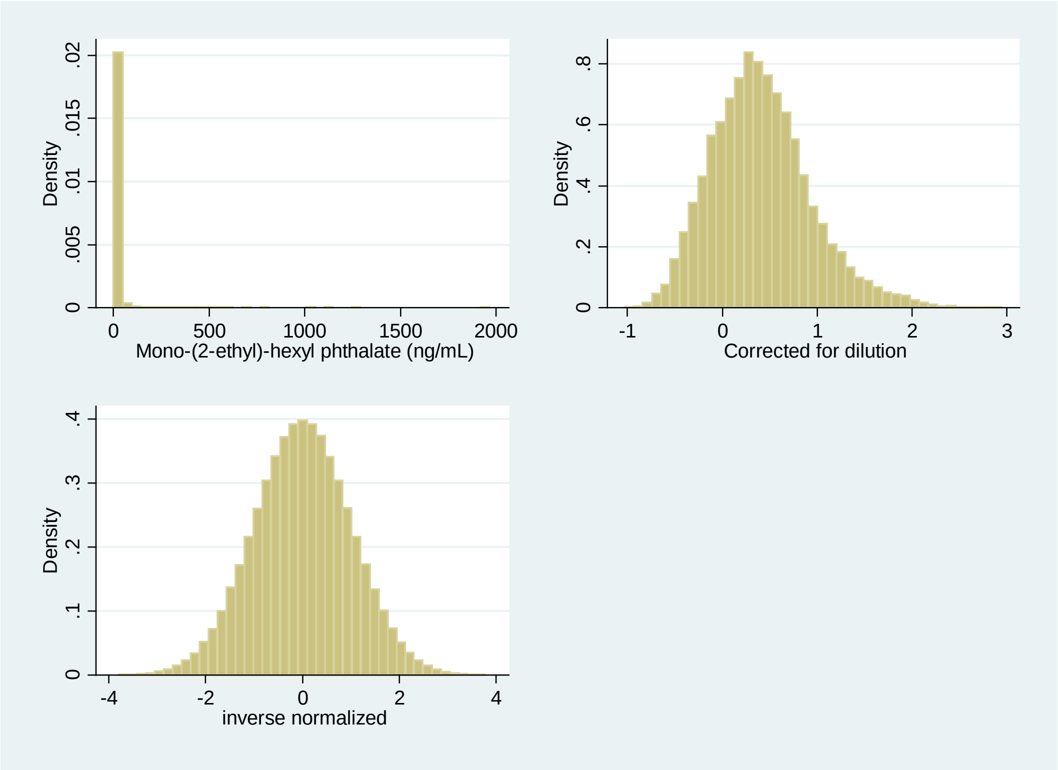

Figure S5: Mono-isononyl phthalate (MNP)

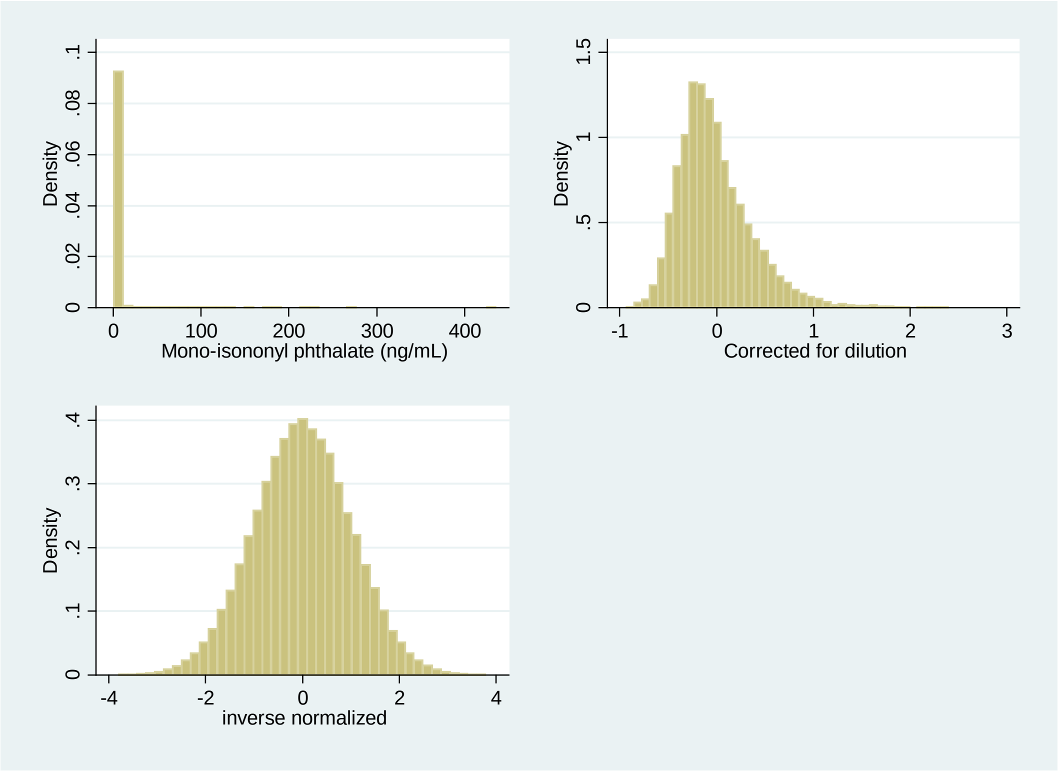

Figure S6: Mono-n-octyl phthalate (MOP)

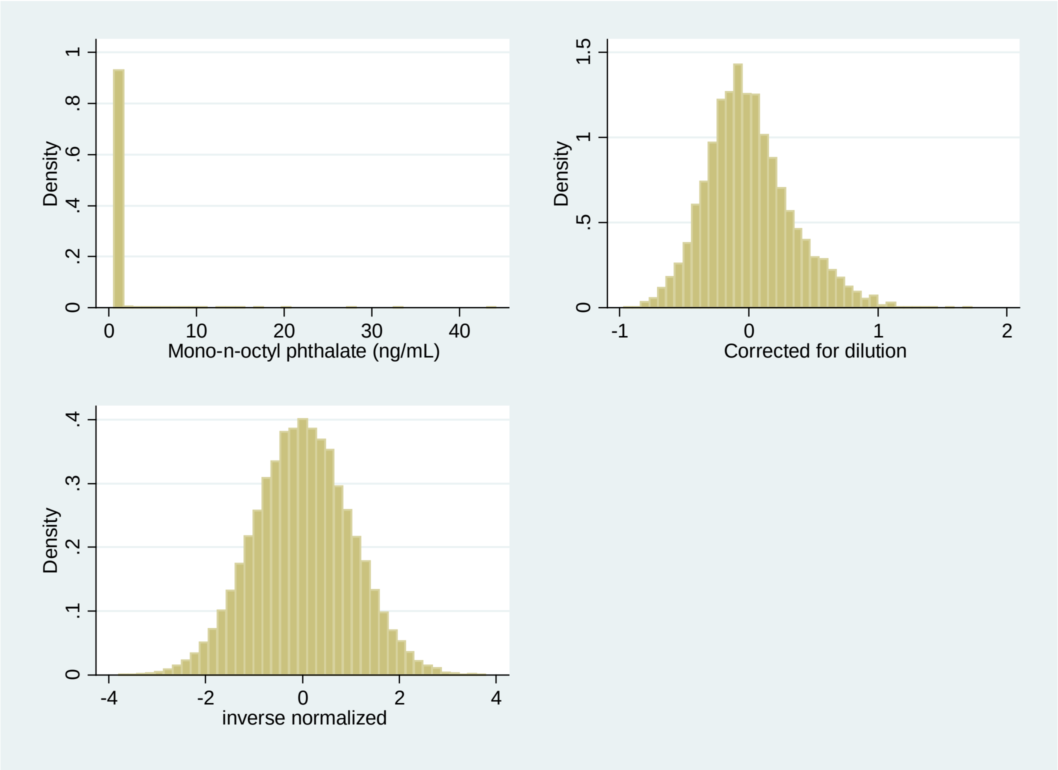

Figure S7: Mono-benzyl phthalate (MBzP)

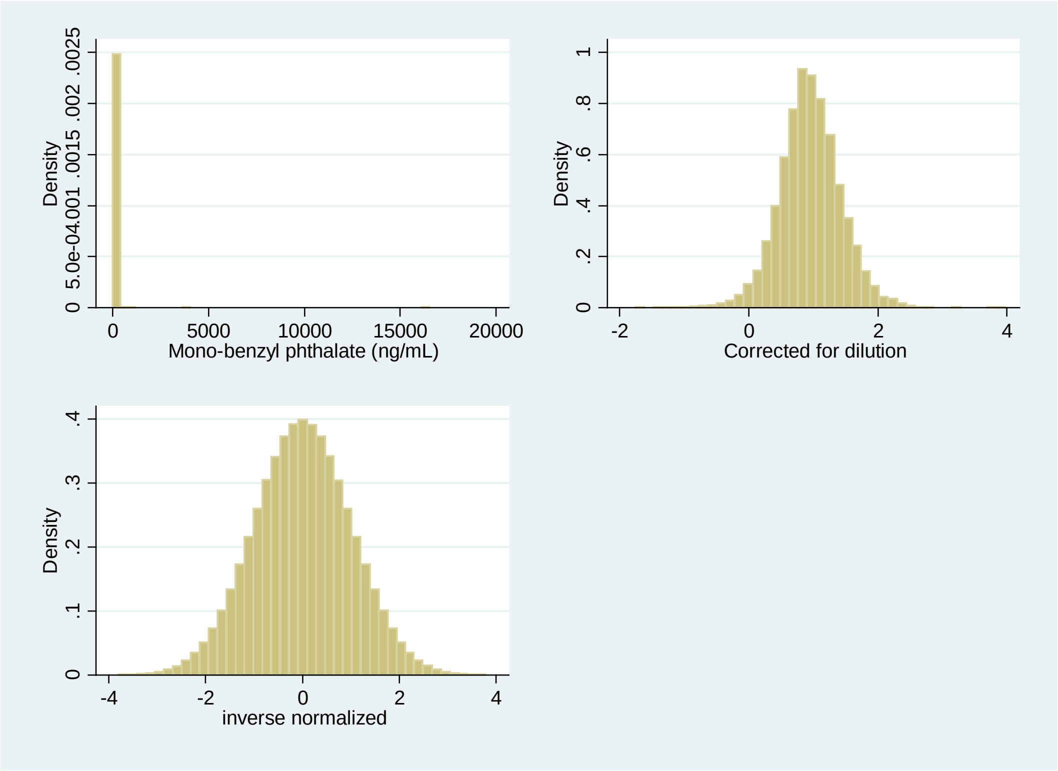

Figure S8: Mono-n-methyl phthalate (MNM)

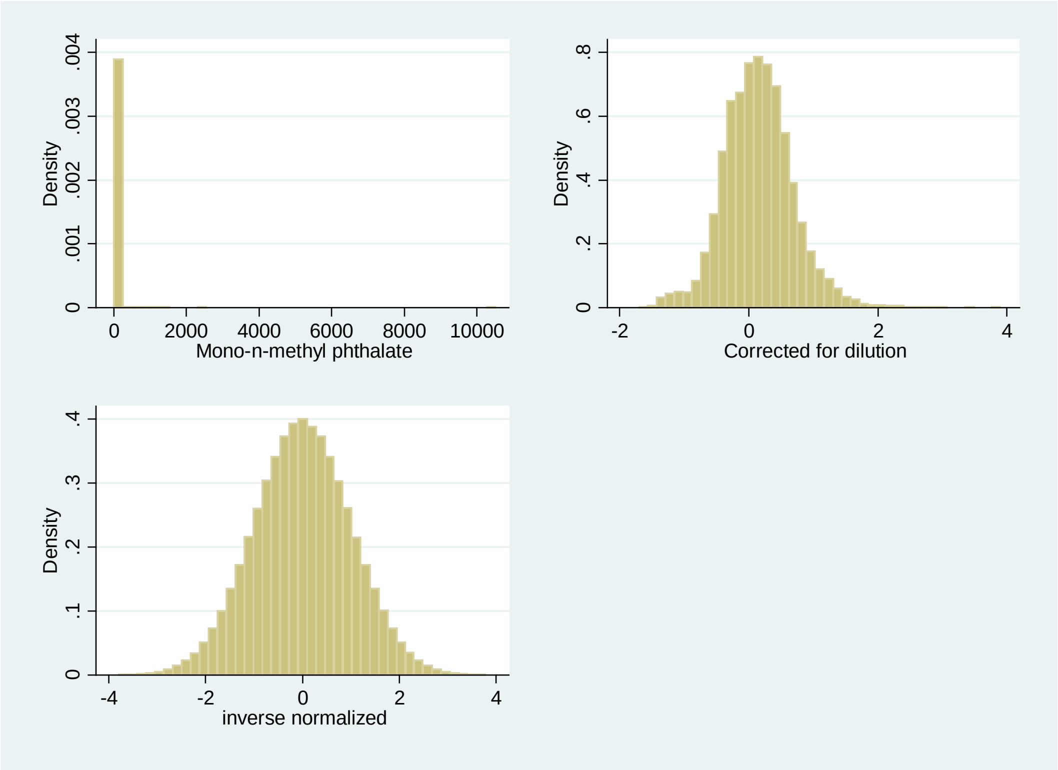

Figure S9: Mono-(3-carboxypropyl) phthalate (MCPP)

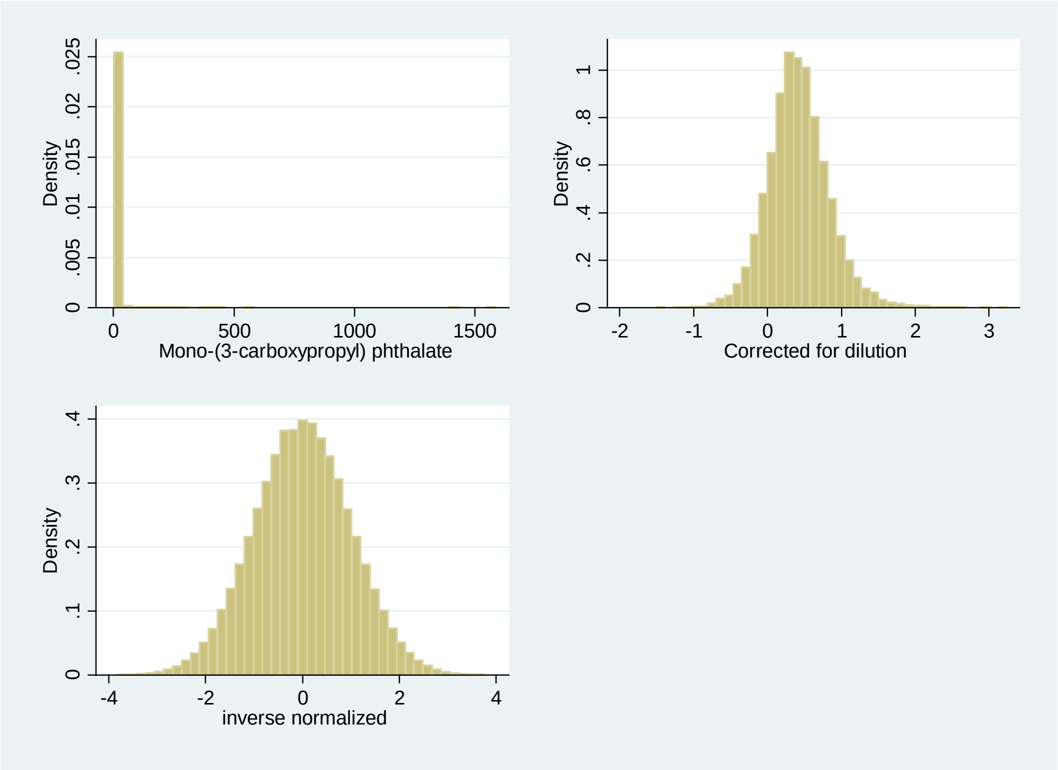

Figure S10: Mono-(2-ethyl-5-hydroxyhexyl) phthalate (MEHHP)

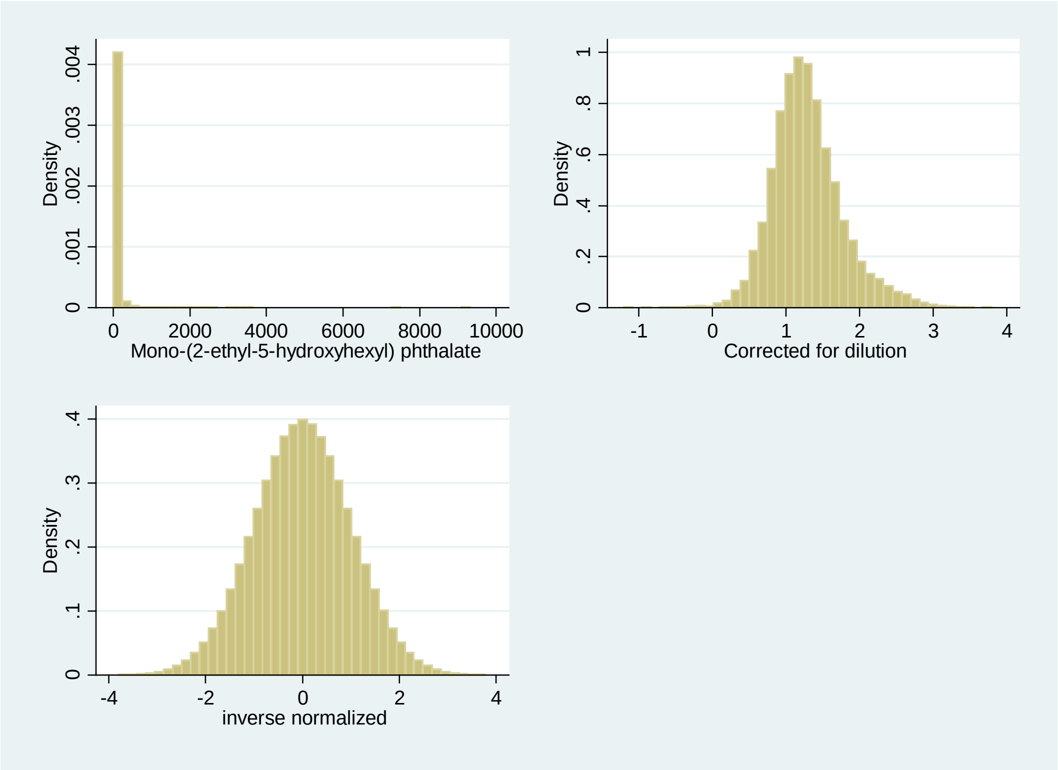

Figure S11: Mono-(2-ethyl-5-oxohexyl) phthalate (MEOHP)

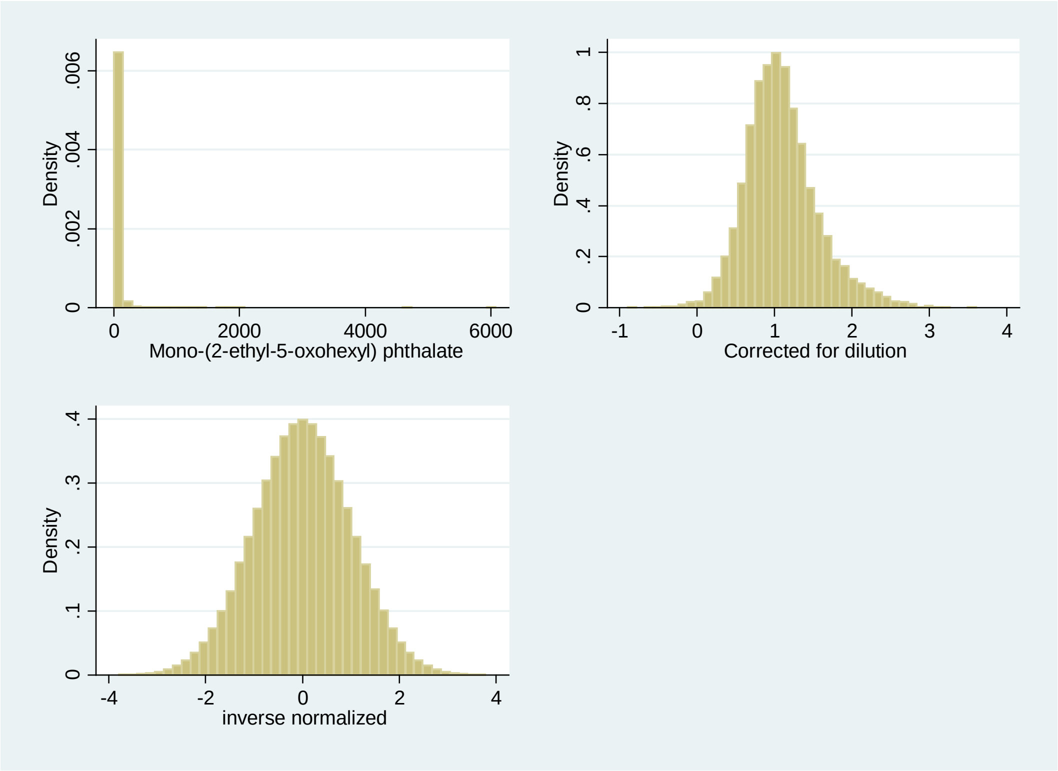

Figure S12: Mono-isobutyl phthalate (MiBP)

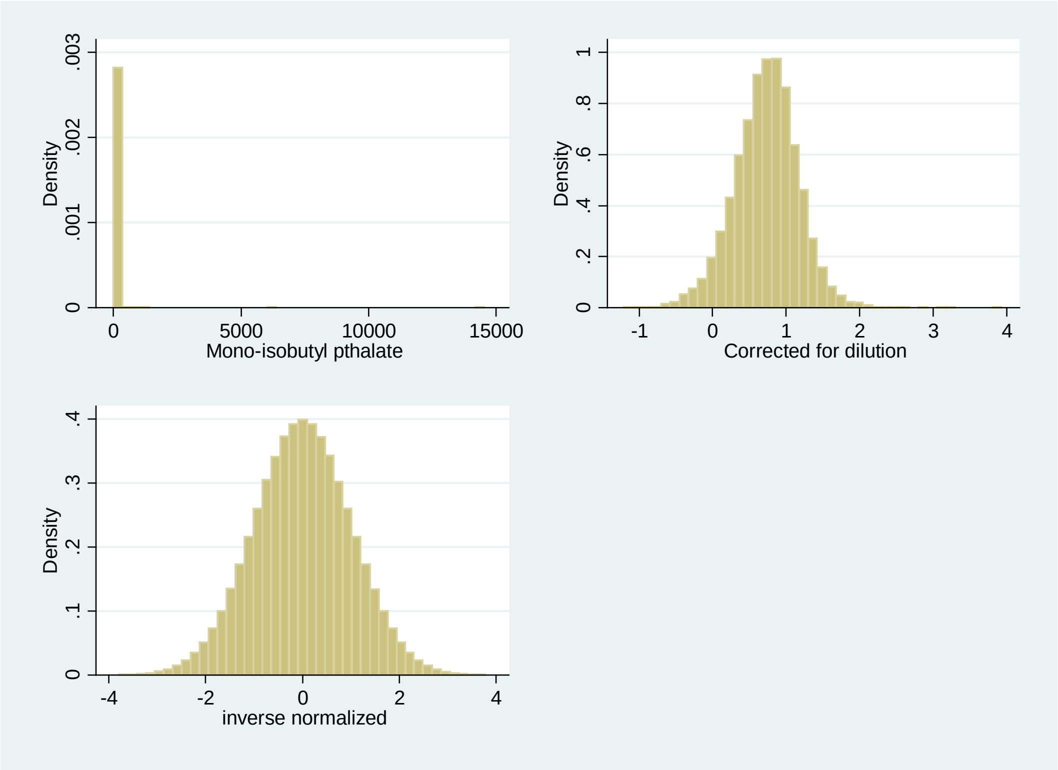

Figure S13: Mono-2-ethyl-5-carboxypentyl phthalate (MECPP)

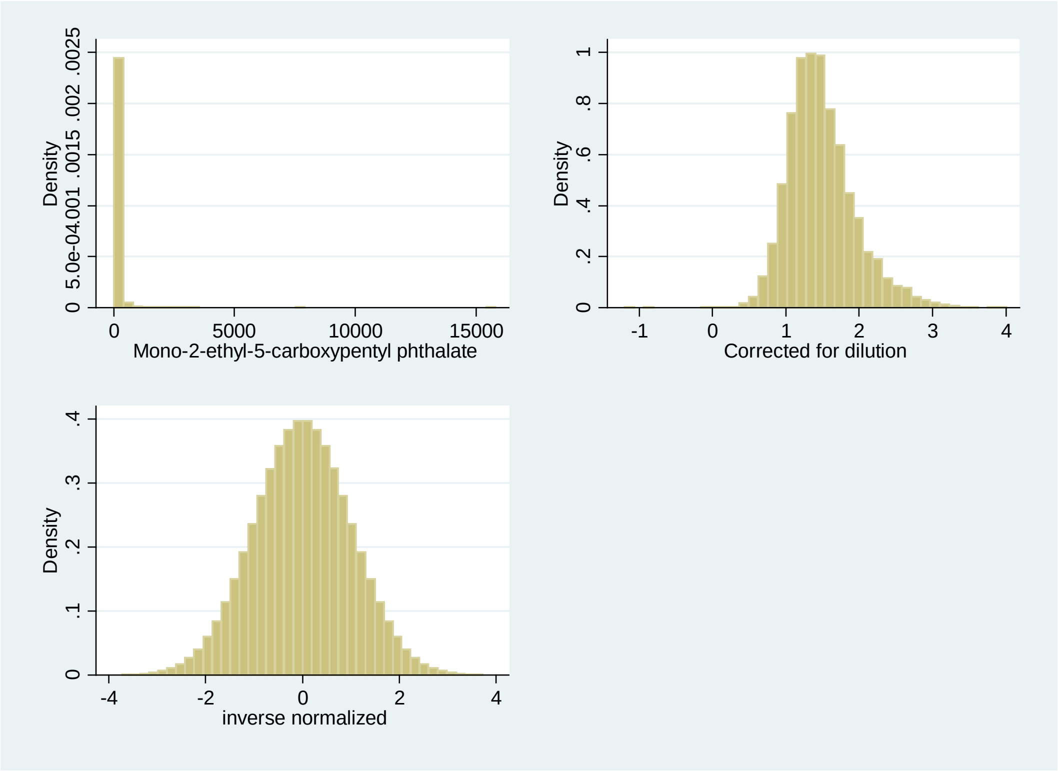

Figure S14: Mono-(carboxynonyl) phthalate (MCNP)

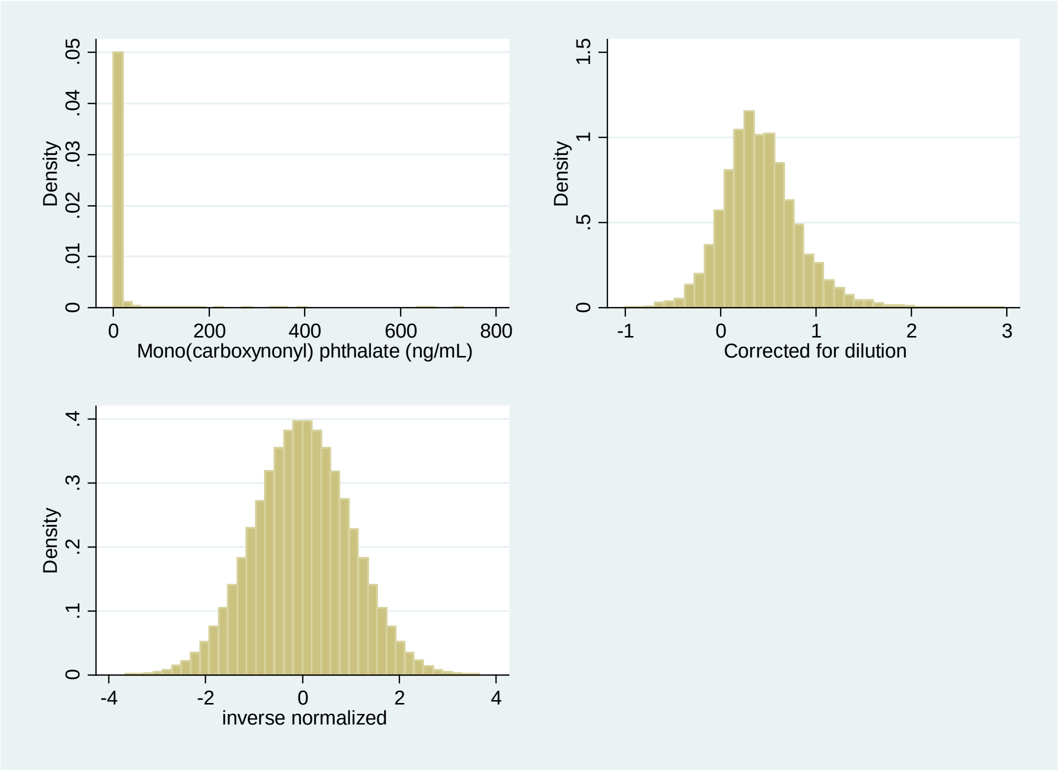

Figure S15: Mono-(carboxyoctyl) phthalate (MCOP)

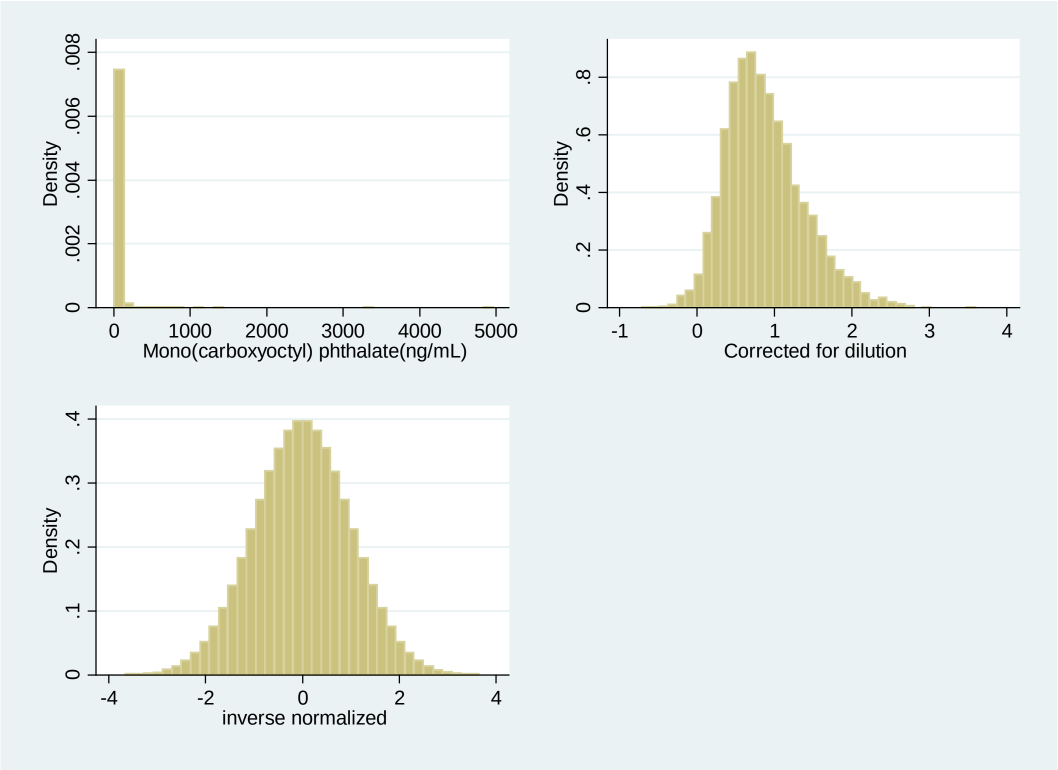

Figure S16: Stata program listing for Monte Carlo simulation of the association of MOP with diabetic retinopathy based on misclassification rates observed in the validation sample

```
program misclasssens
    tempname sim
    postfile `sim' m1 m2 rep coeff secoeff using "misclass.dta", replace
    forvalues m1 = .1120/.1120 { //Misclassification of cases based on validation
       forvalues m2 = 0.0492/0.0492 { //Misclassification of controls based on validation
           display `m1' `","' `m2'
               forvalues i = 1/5000 { //Begin replicates
                   quietly {
                       gen rnddm = runiform()
                       gen tempretinofinal1 = retinofinal if retinofinal==1
                       gen tempretinofinal0 = retinofinal if retinofinal==0
                       replace tempretinofinal1 = 0 if tempretinofinal1!=.&rnddm<`m1'
                       replace tempretinofinal0 = 1 if tempretinofinal0!=.&rnddm<`m2'
                       gen tempretinofinal = tempretinofinal1
                       replace tempretinofinal = tempretinofinal0 if tempretinofinal0!=.
                       //Run full logistic regression model
                       svy: logistic tempretinofinal iph1 iph2 iph3 iph4 iph5 iph6 iph7 iph8 iph9
                       iph10 iph11 iph12 ridageyr male hispmex married dmdeduc2 pir padmetcat a1c
                       hichol lowhdl hitg ht diabdurcat caloriecat obesity
                       mat b = e(b)
                       mat v = e(V)
                       local c = b[1,6]
                       local se = sqrt(v[6,6])
                       post `sim' (`m1') (`m2') (`i') (`c') (`se')
                       drop rnddm tempre*
                   }
                }
        }
     }
     postclose `sim'
end
```

SUPPLEMENTARY TABLES

FOR

ASSOCIATION OF URINARY PHTHALATES WITH SELF-REPORTED EYE AFFLICTION/RETINOPATHY IN INDIVIDUALS WITH DIABETES: NATIONAL HEALTH AND NUTRITION EXAMINATION SURVEY, 2001-2010

Manju Mamtani, MD, Joanne E. Curran, PhD, John Blangero, PhD, Hemant Kulkarni, MD

CONTENTS

This file contains following two tables.

Table	Title	Page
S1	Correlations among urinary phthalate metabolites	2
S2	Final logistic regression model for results shown in Table 3	3

Table S1. Correlations among urinary phthalate metabolites. Each pairwise correlation is shown as Pearson's correlation coefficient and its significance value.

	MBP	MCP	MEP	MEHP	MNP	MOP	MBzP	MNM	MCPP	MEHHP	MEOHP
**MCP**	0.0401										
	0.2041										
**MEP**	0.1694	0.0833									
	<0.0001	0.0083									
**MEHP**	0.2131	0.3328	0.0996								
	<0.0001	<0.0001	0.0016								
**MNP**	0.0313	0.7089	0.0655	0.3526							
	0.321	<0.0001	0.0379	<0.0001							
**MOP**	0.0554	0.8826	0.0877	0.3524	0.7447						
	0.0796	<0.0001	0.0054	<0.0001	<0.0001						
**MBzP**	0.4808	-0.0347	0.0963	0.1648	-0.0164	-0.0422					
	<0.0001	0.2714	0.0023	<0.0001	0.604	0.1816					
**MNM**	0.1633	0.272	0.1715	0.0991	0.2574	0.2735	0.0881				
	<0.0001	<0.0001	<0.0001	0.0017	<0.0001	<0.0001	0.0052				
**MCPP**	0.3436	-0.0023	0.0046	0.1477	0.2213	-0.0013	0.2653	0.0866			
	<0.0001	0.9411	0.8839	<0.0001	<0.0001	0.9671	<0.0001	0.006			
**MEHHP**	0.3868	0.0553	0.0889	0.5927	0.1055	0.1031	0.3461	0.0823	0.3194		
	<0.0001	0.0796	0.0048	<0.0001	0.0008	0.0011	<0.0001	0.0091	<0.0001		
**MEOHP**	0.3844	0.0429	0.0938	0.5936	0.1017	0.0965	0.3428	0.0786	0.3265	0.9598	
	<0.0001	0.1748	0.0029	<0.0001	0.0013	0.0022	<0.0001	0.0127	<0.0001	<0.0001	
**MiBP**	0.3916	-0.0577	0.1025	0.0154	-0.0506	-0.1385	0.2071	0.1067	0.1643	0.2081	0.1781
	<0.0001	0.0678	0.0011	0.6268	0.1092	<0.0001	<0.0001	0.0007	<0.0001	<0.0001	<0.0001

Table S2. Results of final logistic regression model (Model 16 in Table 3)

Covariate	OR	95% CI	P
Mono-n-butyl (MBP)	0.96	0.75 - 1.23	0.753
Mono-cyclohexyl (MCP)	0.80	0.45 - 1.41	0.440
Mono-ethyl (MEP)	1.00	0.77 - 1.29	0.992
Mono-(2-ethyl)-hexyl (MEHP)	0.78	0.57 - 1.06	0.110
Mono-isononyl (MNP)	0.82	0.42 - 1.61	0.565
Mono-n-octyl (MOP)	2.02	1.22 - 3.35	0.007
Mono-benzyl (MBzP)	1.13	0.84 - 1.52	0.401
Mono-n-methyl (MNM)	0.81	0.60 - 1.09	0.170
Mono-(3-carboxypropyl) (MCPP)	0.93	0.71 - 1.21	0.579
Mono-(2-ethyl-5-hydroxyhexyl) (MEHHP)	0.78	0.32 - 1.90	0.579
Mono-(2-ethyl-5-oxohexyl) (MEOHP)	1.82	0.72 - 4.60	0.201
Mono-isobutyl (MiBP)	1.18	0.88 - 1.59	0.264
Age	1.00	0.98 - 1.02	0.645
Male sex	1.13	0.60 - 2.12	0.696
Hispanic/Mexican race	0.87	0.53 - 1.43	0.590
Marital status	1.05	0.65 - 1.67	0.851
Educational attainment	0.86	0.69 - 1.08	0.200
Poverty income ratio	1.06	0.88 - 1.27	0.552
Categories of physical activity score	0.81	0.55 - 1.20	0.290
Strata based on glycated hemoglobin	1.08	0.79 - 1.49	0.612
High total serum cholesterol	0.87	0.43 - 1.78	0.703
Low HDL cholesterol	0.54	0.33 - 0.89	0.017
High serum triglycerides	0.99	0.70 - 1.40	0.947
Hypertension	1.09	0.75 - 1.60	0.641
Categories od duration of diabetes	1.95	1.62 - 2.33	0.000
Categories ot toal calorie intake	0.84	0.68 - 1.05	0.130
Obesity	0.96	0.59 - 1.55	0.854
